# Corrigendum: Glucose-Regulated Protein 78 Signaling Regulates Hypoxia-Induced Epithelial–Mesenchymal Transition in A549 Cells

**DOI:** 10.3389/fonc.2020.615415

**Published:** 2020-12-15

**Authors:** Ling-Ling Sun, Chang-Ming Chen, Jue Zhang, Jing Wang, Cai-Zhi Yang, Li-Zhu Lin

**Affiliations:** Integrative Cancer Centre, The First Afﬁliated Hospital of Guangzhou University of Chinese Medicine, Guangzhou, China

**Keywords:** lung cancer, lung adenocarcinoma, epithelial mesenchymal transition, hypoxia, glucose-regulated protein 78, GRP78

In the original article, there was a mistake in **Figure 1** and **2** as published. Category I images were duplicated. The corrected **Figure 1** and **2** appear below.

**Figure 1:**
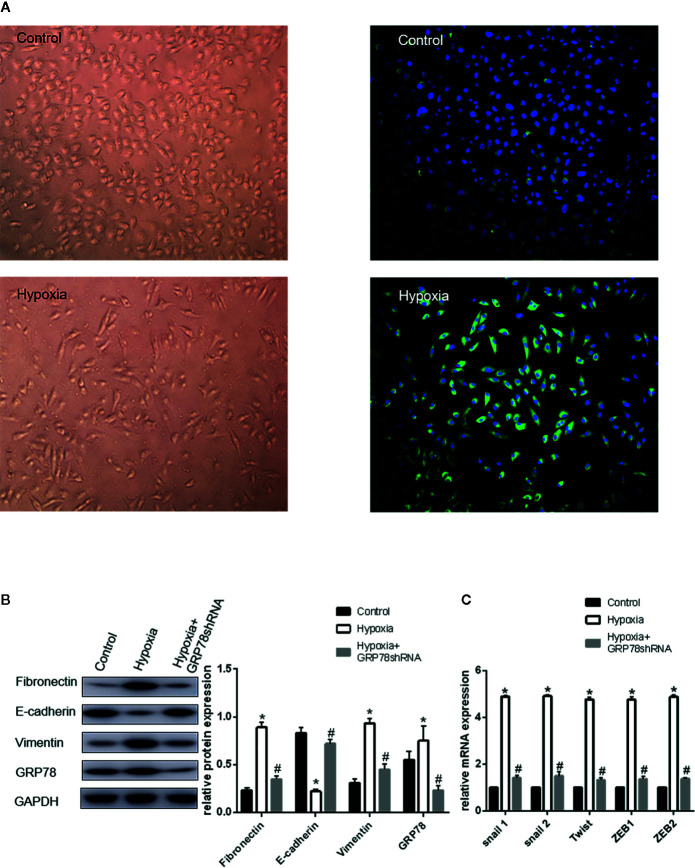
Up-regulation of GRP78 plays an important role in hypoxia-induced EMT in A549 cells. **(A)** A549 cells acquire spindle-shaped mesenchymal morphology after 72 h of 2% O_2_ hypoxia (left, 100×). GRP78 (green fluorescence) is highly expressed in A549 cells with spindle-shaped mesenchymal morphology (right, 100×). **(B)** EMT-related markers (E-cadherin, Vimentin and Fibronectin) and GRP78 were examined by Western blot analysis (left). GAPDH was used as internal control. The protein relative value (GAPDH) is plotted in the right panel (mean ± SD in three separate experiments). *P < 0.05, compared with A549 cells under the condition of normal oxygen, the expression of E-cadherin decreases, while those of Vimentin and Fibronectin increase in A549 cells under hypoxia (2% O_2_ 72 h). The expression of GRP78 also increases in A549 cells under hypoxia. #*P* < 0.05, compared with the A549 cells under the condition of hypoxia; the expression of E-cadherin increases, and those of Vimentin and Fibronectin decrease in GRP78 knockdown A549 cells under hypoxia. **(C)** EMT-related genes including Snail1, Snail2, Twist, ZEB1, and ZEB2 were examined by real-time quantitative PCR; mRNA expression relative value (control group) is plotted (mean ± SD in three separate experiments). *P < 0.05, compared with A549 cells in the control group, the mRNA expression levels of EMT-related genes including Snail1, Snail2, Twist, ZEB1, and ZEB2 increase under hypoxic condition (2% O_2_ 72 h); #*P* < 0.05, compared with A549 cells under the condition of hypoxia, the mRNA expression levels of EMT-related genes decrease in GRP78 knockdown A549 cells under hypoxia.

**Figure 2:**
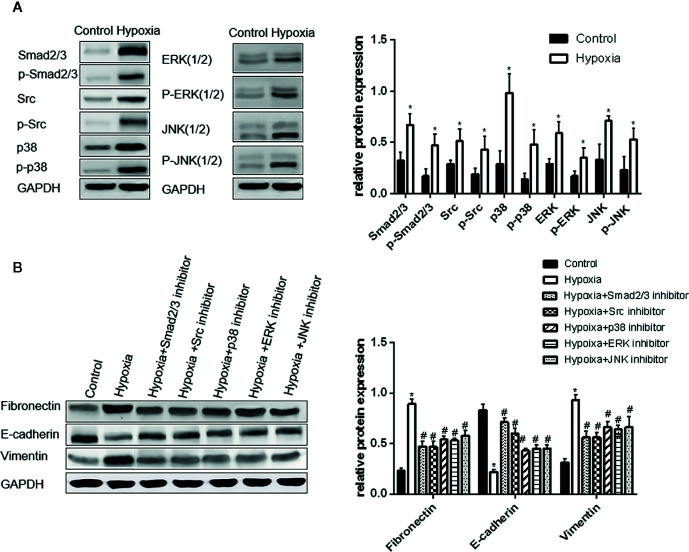
Activation of Smad2/3, Src, p38, ERK, and JNK is important in hypoxia-induced EMT in A549 cells. **(A)** Smad2/3, Src, p38, ERK, JNK, and their phosphorylated forms were examined by Western blot analysis (left). GAPDH was used as internal control. The protein relative value (GAPDH) is plotted in the right panel (mean ± SD in three separate experiments). *P < 0.05, compared with A549 cells in the normal oxygen environments, the Smad2/3, Src, and MAPK proteins of A549 cells are highly regulated and activated in hypoxia environments. **(B)** EMT markers were examined by Western blot analysis (left). GAPDH was used as internal control. The protein relative value (GAPDH) is plotted in the right panel (mean ± SD in three separate experiments). *P < 0.05, compared with A549 cells in the normal oxygen environments, the EMT process of A549 cells under hypoxia is activated; #*P* < 0.05, compared with A549 cells in the hypoxia environments, the EMT process of A549 cells under hypoxia is inhibited separately by Smad2/3, Src, p38, ERK, and JNK inhibitors. The expression levels of Fibronectin and Vimentin decrease, and that of E-cadherin increases.

The authors apologize for this error and state that this does not change the scientific conclusions of the article in any way. The original article has been updated.

